# Clinical Efficacy and Safety of Xinmailong Injection for the Treatment of Chronic Heart Failure: A Meta-Analysis

**DOI:** 10.3389/fphar.2018.00810

**Published:** 2018-07-27

**Authors:** Xiaohua Lu, Lu Zhang, Jiabo Wang, Honghong Liu, Haotian Li, Houqin Zhou, Rongrong Wu, Yuxue Yang, Jianxia Wen, Shizhang Wei, Xuelin Zhou, Yanling Zhao, Xiaohe Xiao

**Affiliations:** ^1^Department of Pharmacy, 302 Military Hospital of China, Beijing, China; ^2^Pharmacy College, Chengdu University of Traditional Chinese Medicine, Chengdu, China; ^3^China Military Institute of Chinese Medicine, 302 Military Hospital of China, Beijing, China; ^4^International Center for Liver Disease Treatment, 302 Military Hospital of China, Beijing, China

**Keywords:** *Periplaneta americana* L., Xinmailong Injection, chronic heart failure, clinical efficacy and safety, meta-analysis

## Abstract

**Background:** Chronic heart failure (CHF) is one of the most stubborn cardiovascular disease. Xinmailong (XML), a bioactive fraction extracted from *Periplaneta americana* L., has been commonly used for CHF treatment in China. However, there is few comprehensive evaluation for the clinical efficacy and safety of XML for CHF.

**Objectives:** We aimed to evaluate the beneficial and adverse effects of Xinmailong Injection (XMLI) on CHF treatment with the use of meta-analysis.

**Methods:** In accordance with the Cochrane Handbook and transparent reporting of systematic reviews and meta-analysis protocol (CRD42018087091), seven English and Chinese electronic databases, including PubMed, EMBASE, Cochrane Library, Chinese National Knowledge Infrastructure (CNKI), Wanfang database, VIP medicine information system and China Biomedical Literature Database (CBM), were searched to retrieve potential randomized controlled trials (RCTs) before November 2017. The eligible trials were evaluated for methodological quality. The main outcome measures were analyzed with RevMan 5.3 software.

**Results:** 26 RCTs involving 3447 participants were subjected to meta-analysis. The total effective rate was improved by XMLI plus conventional therapy (OR 3.10, 95% CI 2.47–3.90, *P* < 0.00001). When compared to the conventional treatment alone, the combination of XMLI and conventional treatment increased left ventricular ejection fraction (LVEF, MD 4.93, 95% CI 3.96–5.89, *P* < 0.00001) and 6-min walking distance (6-MWD, MD 46.76, 95% CI 32.51 to 61.01, *P* < 0.00001), and decreased left ventricular end-diastolic diameter (LVEDD, MD −4.73, 95% CI−5.64 to−3.83, *P* < 0.00001), serum brain natriuretic peptide (BNP, MD −149.59, 95% CI −211.31 to −87.88, *P* < 0.00001) and N-terminal pro-brain natriuretic peptide (NT-proBNP, MD −322.35, 95% CI −517.87 to −126.83, *P* = 0.001). However, the frequency and severity of adverse effects was similar between these two different medications. Poor methodological quality and the limitations also existed in this study.

**Conclusions:** The combinational use of XMLI on conventional treatment may exert better therapeutic effects on improving cardiac function in CHF patients, indicating that XMLI was suggested to be considered during the conventional treatment of CHF. High-quality and large scale RCTs are still required to confirm the impacts of XMLI.

## Introduction

Chronic heart failure (CHF) has become one of the most prevalent cardiovascular disease, affecting about 26 million people worldwide (Ambrosy et al., 2014). It is the leading cause of death in China with a growing number of aging population (Qiu and Wang, 2017). About 0.9% of Chinese population (around 13 billion) are suffering from this disease (Fu et al., 2011). High health care utilization and poor prognosis remain challenging features of this complicated and multifaceted syndrome(Page, 2015).

Although the pathogenesis of CHF has not been fully clarified, it is widely acknowledged that CHF is caused by a structural and/or functional cardiac abnormality, leading to reduced, mid-range and preserved ejection fraction based on left ventricular ejection fraction (LVEF) (Butler, 2012; Ponikowski et al., 2016). The patients with hypertension, coronary heart disease, hyperlipemia, diabetes, and/or myocardial infarction face high risks of HF (Huang, 2015).

The treatment strategies for patients with CHF are complex due to the different pathogenesis and complications. All symptomatic patients with heart failure are recommended with different treatments as follows: (1) Angiotensin-converting enzyme inhibitors (ACEIs); (2) Beta-blockers; (3) Mineralocorticoid/aldosterone receptor antagonists (MRAs). Diuretics, angiotensin receptor neprilysin inhibitor, I_f_-channel inhibitor, angiotensin II type I receptor blockers (ARBs), hydralazine and isosorbide dinitrate, digoxin, n-3 polyunsaturated fatty acids (n-3 PUFAs) are also used to treat patients with CHF based on individual signs and symptoms (Yancy et al., 2013; Ponikowski et al., 2016). However, the above treatments for CHF are not satisfying in terms of low clinical efficacy and safety.

Cumulative research on CHF treatments has reported the combination use of western medicine and traditional Chinese medicines (TCMs) such as Shenmai injection, Wenxin keli, Xinmailong (Fu et al., 2010; Ma et al., 2013; Shi L. et al., 2015; Wang et al., 2016), which displayed better clinical efficacy and lower incidence of side effects. Therefore, it is valuable to take TCMs into account for CHF treatment.

Xinmailong injection (XMLI) is a bioactive fraction extracted from *Periplaneta americana* L. (a species of cockroach) (2016). XMLI has polyhydric alcohols, organic acids, alkaloids and other micro constituents, with complex nucleobases and binding amino acids, including inosine, adenosine, pyroglutamic acid and saccharine, as the active constitutes (Jiao et al., 2011, 2012). XMLI combined with western medicine can treat CHF, ischemic cardiomyopathy and coronary heart failure (Wu and HH, 2015; Yang X. Q. et al., 2015). In 2006, XMLI was approved by the China State Food and Drug administration (CFDA) for CHF treatment. However, there is little comprehensive evaluation for the clinical efficacy and safety of XMLI for CHF. Therefore, this meta-analysis aimed to systematically evaluate the therapeutic effect and safety of XMLI in combination with conventional therapy for CHF treatment when compared with conventional therapy alone.

## Materials and methods

The present meta-analysis was performed in accordance with the Preferred Reporting Items for Systematic Reviews and Meta Analyses (PRISMA) guidelines and Cochrane Handbook, and has been registered in Preferred Reporting Items for International Prospective Register of Systematic Reviews (PROSPERO, CRD42018087091).

### Database and search strategies

Seven major electronic databases, including PubMed, EMBASE, Cochrane Library, Chinese National Knowledge Infrastructure (CNKI), Wanfang database, VIP medicine information system and China Biomedical Literature Database (CBM), were searched to retrieve potential reports by three investigators (Xiaohua Lu, Lu Zhang and Houqin Zhou) independently, with the last search conducted on November 2017.

The initial search items were used as follows: “Xinmailong Injection” [Title/Abstract] or “Xinmailong” [Title/Abstract] and “heart failure” [Title/Abstract] or “chronic heart failure” [Title/Abstract] and “randomized controlled trial” [Title/Abstract].

### Inclusion criteria

Three investigators (Xiaohua Lu, Lu Zhang, and Houqin Zhou) worked independently and complied with the PICOS: (1) the “P” for patients diagnosed as CHF based on “2014 Guidelines for the Diagnosis and Treatment of Heart Failure in China” or “2007 Guidelines for the Diagnosis and Treatment of Chronic Heart Failure in China” or “American College of Cardiology/American Heart Association (ACC/AHA) guidelines 2009” or “Treatment of chronic systolic heart failure 2002” or “Internal Medicine 2008” or “Diagnosis and treatment of cardiomyopathy 2007” or “2007 Guidelines for the Diagnosis and Treatment of chronic stable angina pectoris in China” or “2016 ESC Guidelines for the diagnosis and treatment of acute and chronic heart failure”; (2) the “I” for interventions with the combination use of XMLI and conventional therapy (Experimental Group); (3) the “C” for comparison of the conventional therapy alone (Control Group); (4) “O” for outcome measures, including the total effective rate, left ventricular ejection fraction (LVEF), brain natriuretic peptide (BNP), N-terminal pro-brain natriuretic peptide (NT-Pro BNP), left ventricular end-diastolic dimension (LVEDD) and 6-min walking distance (6 MWD) as well as adverse effect. In addition, the type of studies was randomized controlled trials (RCTs).

Those subjects who underwent the significant relief of clinical symptoms and signs of CHF and experienced 2-level of improvement in cardiac function were defined as “remarkable effect” according to New York Heart Association (NYHA) grading. Likewise, those subjects who underwent the relief of clinical symptoms and signs of CHF and experienced 1-level of improvement in cardiac function were considered to be “valid,” otherwise “invalid.” The number of remarkable effect and valid cases together was the total effective rate.

### Exclusion criteria

Three investigators (Xiaohua Lu, Lu Zhang, and Houqin Zhou) worked independently using the following exclusion criteria: (1) animal studies or non-randomized controlled clinical trials; (2) publications with incomplete data or the data from an identical clinical trial; (3) unavailable or incorrect or no relevant data for meta-analysis; (4) patients with acute heart failure, ischemic cardiomyopathy, pulmonary heart disease, severe liver, and kidney diseases; (5) any other TCM or therapies in experimental or control group during the treatment.

### Data extraction

Three investigators (Xiaohua Lu, Lu Zhang, and Houqin Zhou) independently conducted literature screening and data extraction according to the same inclusion and exclusion criteria. The extracted data included study title, author, year of publication, sample size, diagnosis standard, methodological information, treatment process, interventions, outcome measurements, NYHA classification, and adverse effects. Disagreements were resolved by discussion through the third party (Yuxue Yang) and a consensus was reached finally.

### Quality assessment

Based on Cochrane Handbook for Systematic Review of Interventions, the methodological quality of the included trials was assessed to address the following seven criteria: random sequence generation (selection bias), allocation concealment (selection bias), blinding of participants and personnel (performance bias), blinding of outcome assessments (detection bias), incomplete outcome data (attrition bias), selective outcome reporting (reporting bias), and other sources of bias. The risk of bias was categorized as low, unclear, or high. Trials were categorized as low risk of bias when they met all the criteria, whereas those that met none were classified as high risk of bias. Others were unclear risk of bias if there was insufficient information to make a judgment. Any disagreement was settled by discussion with the third author (Yuxue Yang).

### Data analysis

RevMan 5.3 software (http://tech.cochrane.org/revman/download) from the Cochrane Collaboration was utilized to perform the meta-analysis. Dichotomous data were expressed as the Odds ratio (OR) with 95% confidence intervals (95% CI), whereas continuous variables were expressed as mean difference (MD) with 95% confidence intervals (95% CI). Pooled analyses were calculated using fixed-effect models if *P* ≥ 0.10 and *I*^2^ ≤ 50%, indicating low heterogeneity; whereas a random-effect model was applied in case of significant heterogeneity (*P* < 0.10 and *I*^2^ > 50%).

## Results

### Description of the included trials

**Figure 1:**
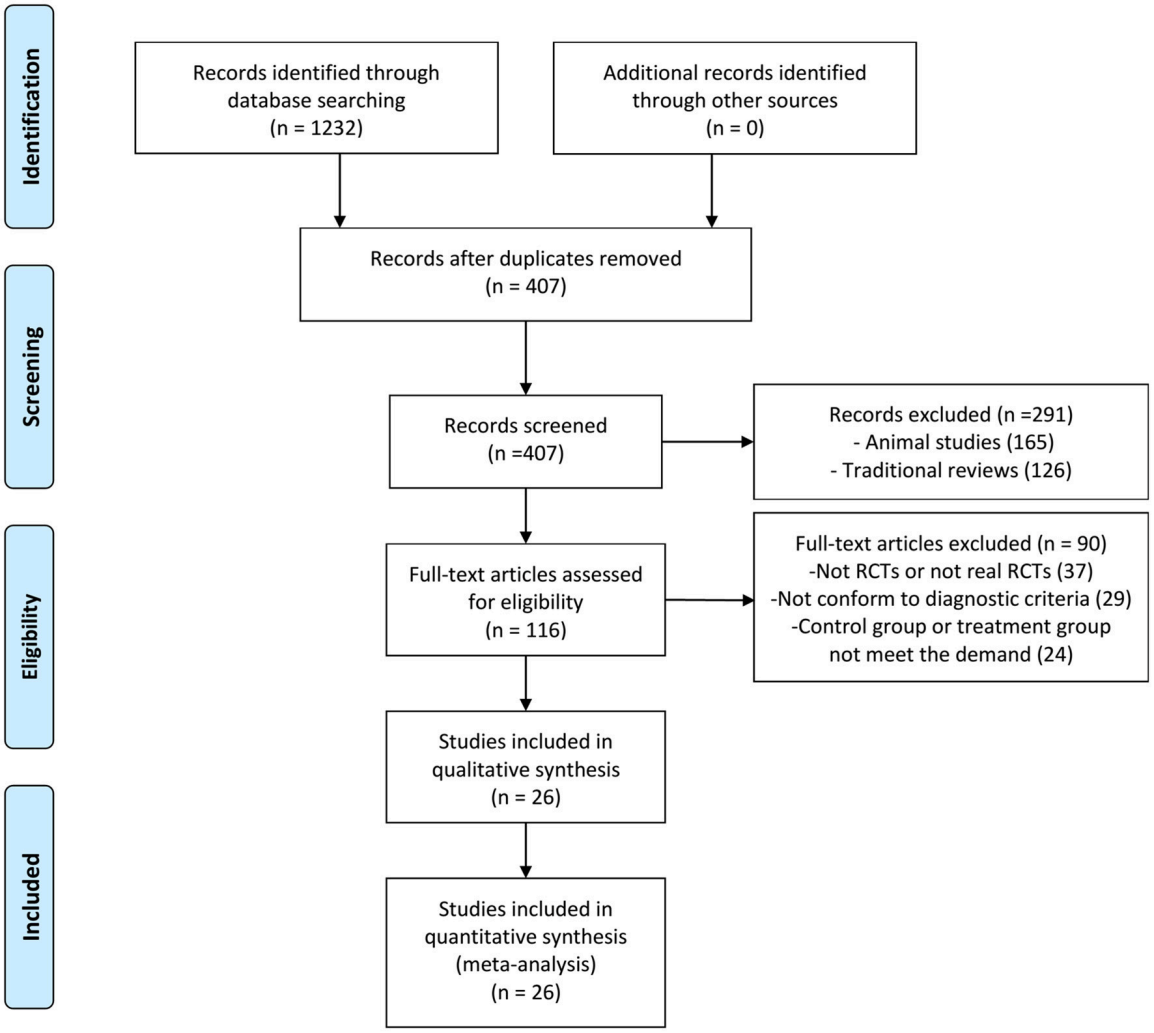
Flow diagram of the article selection for this study. RCTs, Randomized controlled trials.

One thousand two hundred and thirty-two articles were potentially retrieved after the primary search from the seven Chinese and English databases. Eight hundred and twenty-five articles were excluded because of duplicate collections. Two hundred and ninety-one publications were further excluded because they were non-clinical studies. Ninety articles were excluded due to the following reasons: not RCTs or not real RCTs, without confirmed diagnostic criteria, control group or treatment group not meet the demand. Finally, 26 RCTs, published from 2010 to 2017, were included as shown in Figure 1 (Zhao et al., 2010; Yang et al., 2012, 2014; Huang et al., 2013; Lu et al., 2013, 2015; Peng et al., 2014; Li and Li, 2015; Shi H. R. et al., 2015; Wu, 2015; Xue et al., 2015; Yang M. et al., 2015; Yuan et al., 2015; Zhang, 2015; Guo and Ren, 2016; Han and Guo, 2016; Li et al., 2016; Xu and Xu, 2016; Fan et al., 2017; Quan and Miu, 2017; Qu et al., 2017; Shen et al., 2017; Si, 2017; Wang, 2017; Ye et al., 2017; Yu et al., 2017).

All these trials were conducted in China and published in Chinese. A total of 3,447 participants (1,781 cases in the experimental group, and 1,666 cases in the control group) were included in these 26 eligible trials. Participants were divided into experimental (XMLI in combination with conventional therapy) and control group (conventional therapy) with no significant difference between these two groups in general information (Table 1). All the XMLI were from Yunnan Teng yao Pharmaceutical Co., Ltd.

**Table 1:** Characteristics of the included studies.

**Study**	**Indication**	**Diagnosis Standard**	**NYHA**	**Sample Size (E/C)**	**Age (Year)**	**Male/Female (E/C)**	**Course of disease (Year)**	**Intervention**	**Duration (day)**	**Endpoints**
								**Experimental Group**		
Shi H. R. et al., 2015	CHD	2014 Guidelines for the Diagnosis and Treatment of Heart Failure in China	NA	58/58	E:56.20 ± 8.74 C:55.6 ± 9.18	E:28/30 C:29/29	NA	XMLI 5 mg/kg bid+ Control	5	BNP, LVEF, TC, TG, HDL-C, LDL-C, UA, blood glucose, hsCRP, VEGF
Lu et al., 2015	CHF	2007 Guidelines for the Diagnosis and Treatment of Chronic Heart Failure in China	NA	36/36	E:61.2 ± 5.9 C:62.3 ± 5.5	E:23/13 C:24/12	NA	XMLI 5 mg/kg bid+ Control	5	the total effective rate, BNP, LVEF, 6-WMD, adverse effect
Guo and Ren, 2016	CHF	2007 Guidelines for the Diagnosis and Treatment of Chronic Heart Failure in China	II IV	52/52	E:69 ±8 C:68 ± 5	E:27/25 C:28/24	E:8.3 ± 6.0 C:8.3 ± 6.1	XMLI 6 mL bid+ Control	10	the total effective rate, BNP, LVEF, LVEDD, 6-WMD, adverse effect
Han and Guo, 2016	CHF	American College of Cardiology/ American Heart Association (ACC/AHA) guidelines 2009	NA	147/136	E:79 ± 11 C:77 ± 12	E:109/27 C:122/25	NA	XMLI 4 mL bid+ Control	14	the total effective rate, BNP, LVEF
Peng et al., 2014	CHF	Treatment of chronic systolic heart failure 2002	II IV	56/56	E:71.1 ± 2.8 C:70.2 ± 2.6	E:32/24 C:30/26	NA	XMLI 4 mL bid+ Control	14	LVEF, LVEDD, 6-WMD, CO, SV, LVS, LVESV, LVEDV, HR, life quality score, adverse effect
Yang et al., 2014	CHF	2007 Guidelines for the Diagnosis and Treatment of Chronic Heart Failure in China	II III	49/43	E:77.5± 6.2 C:75.2 ± 5.5	E:33/16 C:31/12	NA	XMLI 6 mL bid+ Control	7	6-WMD, NT-proBNP, CTnI
Yang et al., 2012	CHF	American College of Cardiology/ American Heart Association (ACC/AHA) guidelines 2009	III IV	57/53	E:79± 10 C:78 ± 11	E:46/11 C:44/9	NA	XMLI 4 mL bid+ Control	14	the total effective rate, BNP, LVEF
Xue et al., 2015	CHF	American College of Cardiology/ American Heart Association (ACC/AHA) guidelines 2009	II III	118/120	E:63.1± 9.80 C:63.9 ± 9.01	E:69/46 C:60/60	E:754 days C:865 days	XMLI 5 mg/kg bid+ Control	5	the total effective rate, LVEF, 6-WMD, adverse effect, the total effective rate of Chinese medical syndrome efficacy, scores for Chinese medical symptoms
Wu, 2015	CHF	2007 Guidelines for the Diagnosis and Treatment of Chronic Heart Failure in China	NA	25/25	E:65± 4.5 C:66 ± 5.5	E:15/10 C:13/12	NA	XMLI 5 mg/kg bid+ Control	5	the total effective rate, NT-proBNP, adverse effect
Zhao et al., 2010	CHF	2007 Guidelines for the Diagnosis and Treatment of Chronic Heart Failure in China	III IV	59/58	E:63-82 C:60-83	E:26/33 C:30/28	NA	XMLI 5 mg/kg bid+ Control	10	the total effective rate, NT-proBNP, LVEF, metabolic equivalent of energy, adverse effect
Li and Li, 2015	CHF	American College of Cardiology/ American Heart Association (ACC/AHA) guidelines 2009	II IV	35/30	E:62± 10 C:58 ± 8	E:20/15 C:18/12	NA	XMLI 8 mL qd+ Control	15	the total effective rate, LVEF, LVEDD
Xu and Xu, 2016	CHF	2014 Guidelines for the Diagnosis and Treatment of Heart Failure in China	NA	76/76	NA	E:50/26 C:48/28	NA	XMLI 5 mg/kg bid+ Control	14	the total effective rate, LVEF, LVEDD, 6-WMD, LVESD
Zhao et al., 2010	CHF	2007 Guidelines for the Diagnosis and Treatment of Chronic Heart Failure in China	IV	131/112	NA	152/91	NA	XMLI 5-10mg/kg bid+ Control	14	BNP, LVEF, central venous pressure, adverse effect
Yang M. et al., 2015	CHF	Treatment of chronic systolic heart failure 2002	III IV	132/128	E:34-78 C:37-84	E:88/40 C:94/38	NA	XMLI 6 mL bid+ Control	7-14	the total effective rate, LVEF, NT-proBNP, LVEDD, 6-WMD, adverse effect
Yuan et al., 2015	CHF	Internal Medicine 2008	I IV	54/34	E:51.5± 5.6 C:52.3 ± 6.0	E:30/24 C:19/15	E:2.5 ± 2.3 C:2.8 ± 3.1	XMLI 5 mg/kg bid+ Control	5	the total effective rate, NT-proBNP, 6-WMD, adverse effect
Lu et al., 2013	DCM	Diagnosis and treatment of cardiomyopathy 2007	II IV	53/51	E:63.1 ± 7.9 C:62.9 ± 7.6	E:31/22 C:28/23	E:2.5 ± 0.8 C:2.6 ± 0.9	XMLI 8 mL bid+ Control	15	the total effective rate, LVEF, adverse effect
Li et al., 2016	CHF	2007 Guidelines for the Diagnosis and Treatment of Chronic Heart Failure in China	II IV	100/98	E:67.34 C:68.12	E:62/38 C:58/40	NA	XMLI 5 mg/kg bid+ Control	10	the total effective rate, NT-proBNP, LVEF, LVEDD, adverse effect
Huang et al., 2013	CHF	2007 Guidelines for the Diagnosis and Treatment of Chronic Heart Failure in China	III IV	71/46	E:90 ± 4.6 C:89 ± 5.1	E:54/17 C:34/12	E:14 ± 6.8 C:15 ± 5.3	XMLI 8 mL qd+ Control	14	the total effective rate, NT-proBNP, LVEF, adverse effect
Shen et al., 2017	CHD	2007 Guidelines for the Diagnosis and Treatment of chronic stable angina pectoris in China, 2014 Guidelines for the Diagnosis and Treatment of Heart Failure in China	III IV	58/58	E:62.8 ± 7.1 C:61.6 ± 7.8	E:34/24 C:36/22	E:8.3 ± 7.5 C:8.1 ± 7.8	XMLI 4 mL bid+ Control	14	NT-proBNP, LVEF, LVEDD, 6-WMD
Fan et al., 2017	CHF	2007 Guidelines for the Diagnosis and Treatment of Chronic Heart Failure in China	III IV	44/34	E:64.2 ± 7.6 C:63.8 ± 6.8	E:28/16 C:20/14	T:4.5 ± 6.8 C:4.2 ± 6.3	XMLI 5 mg/kg bid+ Control	14	NT-proBNP, LVEF, APN
Ye et al., 2017	CHF	2014 Guidelines for the Diagnosis and Treatment of Heart Failure in China, 2016 ESC Guidelines for the diagnosis and treatment of acute and chronic heart failure	II III	63/63	E:71.31 ± 11.36 C:74.01 ± 13.22	E:39/24 C:43/20	T:9.31 ± 3.25 C:10.51 ± 4.13	XMLI 5 mg/kg bid+ Control	14	NT-proBNP, LVEF, adverse effect, TNF-α, IL-6, VEGF, scores of symptoms and signs for hear failure, adverse effect
Yu et al., 2017	CHF	2014 Guidelines for the Diagnosis and Treatment of Heart Failure in China	NA	70/70	E:70 ± 9 C:68 ± 10	E:38/22 C:43/27	NA	XMLI 5 mg/kg bid+ Control	5	the total effective rate, BNP, LVEF, adverse effect
Qu et al., 2017	CHF	American College of Cardiology/ American Heart Association (ACC/AHA) guidelines 2009	III IV	114/106	E:69 ± 10 C:68 ± 11	E:92/22 C:88/18	NA	XMLI 5-10 mg/kg bid+ Control	14	the total effective rate, BNP, LVEF, 6-WMD, adverse effect
Si, 2017	CHF	2007 Guidelines for the Diagnosis and Treatment of Chronic Heart Failure in China	NA	51/51	E:75.63 ± 8.18 C:74.84 ± 9.76	E:25/26 C:23/28	NA	XMLI 5 mg/kg bid+ Control	14	the total effective rate, BNP, LVEF, adverse effect
Wang, 2017	CHF	2007 Guidelines for the Diagnosis and Treatment of Chronic Heart Failure in China	II III	21/21	E:71.38 ± 9.23 C:71.81 ± 9.92	E:13/8 C:12/9	E:5.19 ± 1.28 C:5.67 ± 1.8	XMLI 5 mg/kg bid+ Control	5	NT-proBNP, LVEF, adverse effect, NYHA classification
Quan and Miu, 2017	CHF	2014 Guidelines for the Diagnosis and Treatment of Heart Failure in China, American College of Cardiology/American Heart Association (ACC/AHA) guidelines 2009	II IV	51/51	E:67.6 ± 10.5 C:65.8 ± 11.4	E:35/16 C:38/13	E:37.8 ± 7.5 C:39.6 ± 8.6	XMLI 5 mg/kg bid+ Control	10	the total effective rate, NT-proBNP, LVEF, 6-WMD, adverse effect

*Notes: all control groups of the included trials were given conventional therapy. NYHA, New York Heart Association; E, experimental group; C, control group; CHD, coronary atherosclerotic heart disease; CHF, chronic heart failure; NA, not applicable; DCM, dilated cardiomyopathy; XMLI, Xinmailong injection; BNP, Brain natriuretic peptide; LVEF, Left ventricular ejection fraction; TC, total cholesterol; TG, triacylglycerol; HDL-C, high density lipoprotein cholesterin; LDL-C, low density lipoprotein cholesterin; UA, Uric acid; hs-CRP, High-sensitivity C-reactive protein; VEGF, vascular endothelial growth; 6-MWD: 6-min walking distance; LVEDD, Left ventricular end-diastolic dimension; LVESD, Left ventricular end systolic diameter; NT-proBNP, N-terminal pro-brain natriuretic peptide; HR, Heart failure; CO, Cardiac output; SV, Stroke output; LVS, Left ventricular end-systolic dimension; LVESV, Left ventricular end-systolic volume; LVEDV, Left ventricular end-diastolic volume; APN, adiponectin; TNF-α, tumor necrosis factor α; IL-6: Interleukin-6*.

### Methodological quality

**Figure 2:**
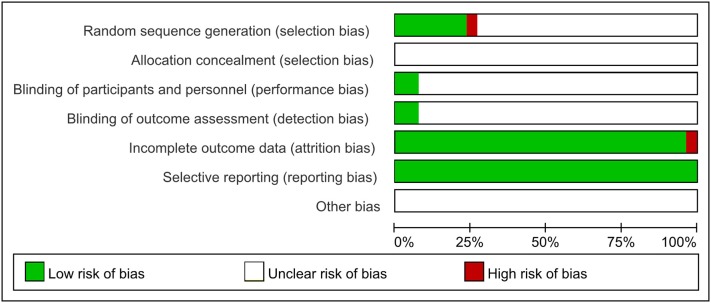
Risk of bias summary.

As shown in Figure 2, among these 26 included articles, 6 trials reported that the methodology was used to generate the allocation sequence, as well as 5 trials for completely random number table (Guo and Ren, 2016; Quan and Miu, 2017; Si, 2017; Wang, 2017; Ye et al., 2017) and 1 trial for randomized complete block design (Xue et al., 2015); While the rest mentioned random sequence generation without specific random method. All these involved trials did not mention the allocation concealment. Two studies mentioned blinding of participants and personnel and outcome assessment (Xue et al., 2015; Shen et al., 2017). One study was published with a high risk with incomplete outcome (Xue et al., 2015). All included trials were published with low risk of selective reporting and without clear statement of other bias.

### Effects of the interventions

#### The total effective rate

**Figure 3:**
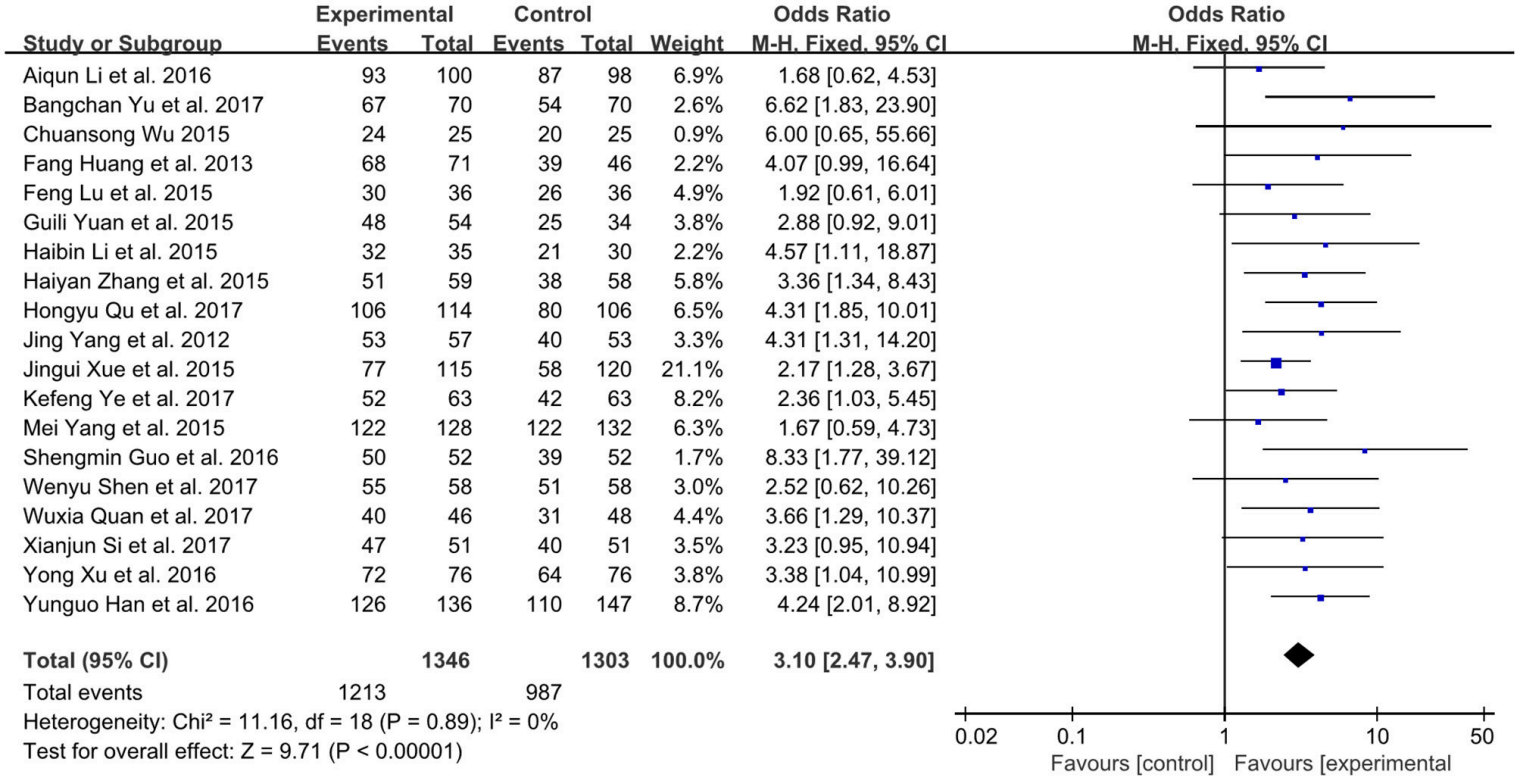
Forest plot of the total effective rate of XMLI plus conventional therapy vs. conventional therapy alone for CHF treatment. *I*^2^ and *P* are the criterion for the heterogeneity test, 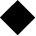 pooled odds ratio, —
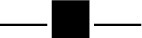
— odds ratio and 95% CI. XMLI, Xinmailong injection; CHF, chronic heart failure; CI, confidence interval.

A total number of 19 studies involving 2,649 participates assessed the clinical improvement based on the total effective rate (Yang et al., 2012; Huang et al., 2013; Li and Li, 2015; Lu et al., 2015; Wu, 2015; Xue et al., 2015; Yang M. et al., 2015; Yuan et al., 2015; Zhang, 2015; Guo and Ren, 2016; Han and Guo, 2016; Li et al., 2016; Xu and Xu, 2016; Quan and Miu, 2017; Qu et al., 2017; Shen et al., 2017; Si, 2017; Ye et al., 2017; Yu et al., 2017). The fixed-effect model was performed because of low heterogeneity (*P* = 0.89, *I*^2^ = 0%). As shown in Figure 3, XMLI in combination with conventional therapy had improved the total effective rate in patients with CHF vs. conventional therapy alone (OR 3.10, 95% CI 2.47 to 3.90, *P* < 0.00001). This indicated that XMLI could significantly increase the clinical efficacy of conventional therapy for CHF.

### Left ventricular ejection fraction (LVEF)

**Figure 4:**
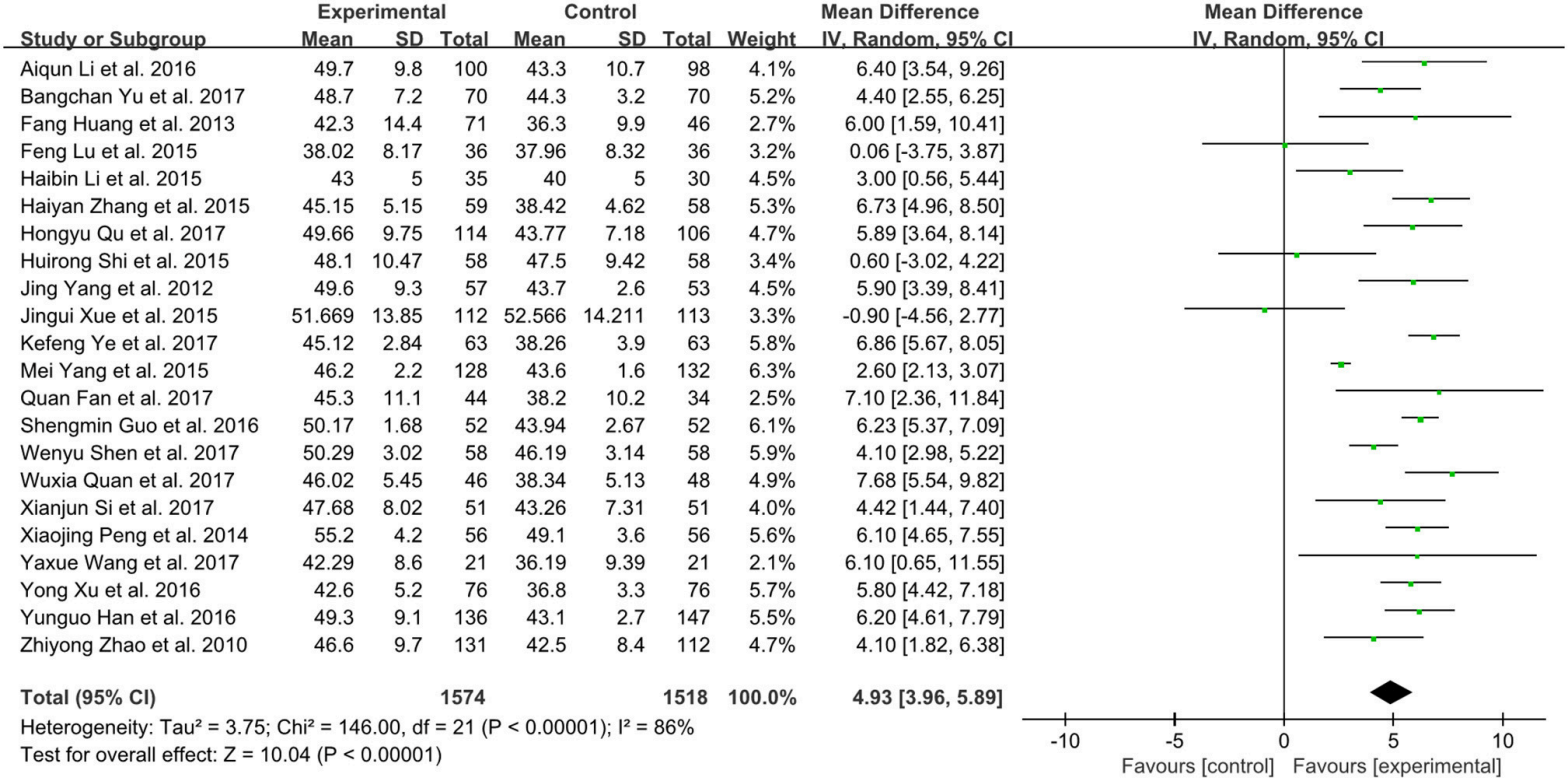
Forest plot of the LVEF of XMLI plus conventional therapy vs. conventional therapy alone for CHF treatment. *I*^2^ and *P* are the criterion for the heterogeneity test, 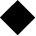 pooled mean difference, —
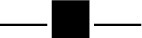
— mean difference and 95% CI. XMLI, Xinmailong injection; CHF, chronic heart failure; CI, confidence interval.

Twenty-two trials in total involving 3,092 patients with CHF assessed the effect of XMLI plus conventional therapy vs. conventional therapy alone in boosting LVEF (Zhao et al., 2010; Yang et al., 2012; Huang et al., 2013; Peng et al., 2014; Li and Li, 2015; Lu et al., 2015; Shi H. R. et al., 2015; Xue et al., 2015; Yang M. et al., 2015; Zhang, 2015; Guo and Ren, 2016; Han and Guo, 2016; Li et al., 2016; Xu and Xu, 2016; Fan et al., 2017; Quan and Miu, 2017; Qu et al., 2017; Shen et al., 2017; Si, 2017; Wang, 2017; Ye et al., 2017; Yu et al., 2017). Random effect model was conducted due to substantial heterogeneity among these trials (*I*^2^ = 86%, *P* < 0.00001). The result of this meta-analysis showed that LVEF value in the experimental group was significantly higher than that of control group (MD 4.93, 95% CI 3.96 to 5.89, *P* < 0.00001), which indicated that XMLI could improve LVEF when compared those in the conventional therapy for CHF (Figure 4).

**Figure 5:**
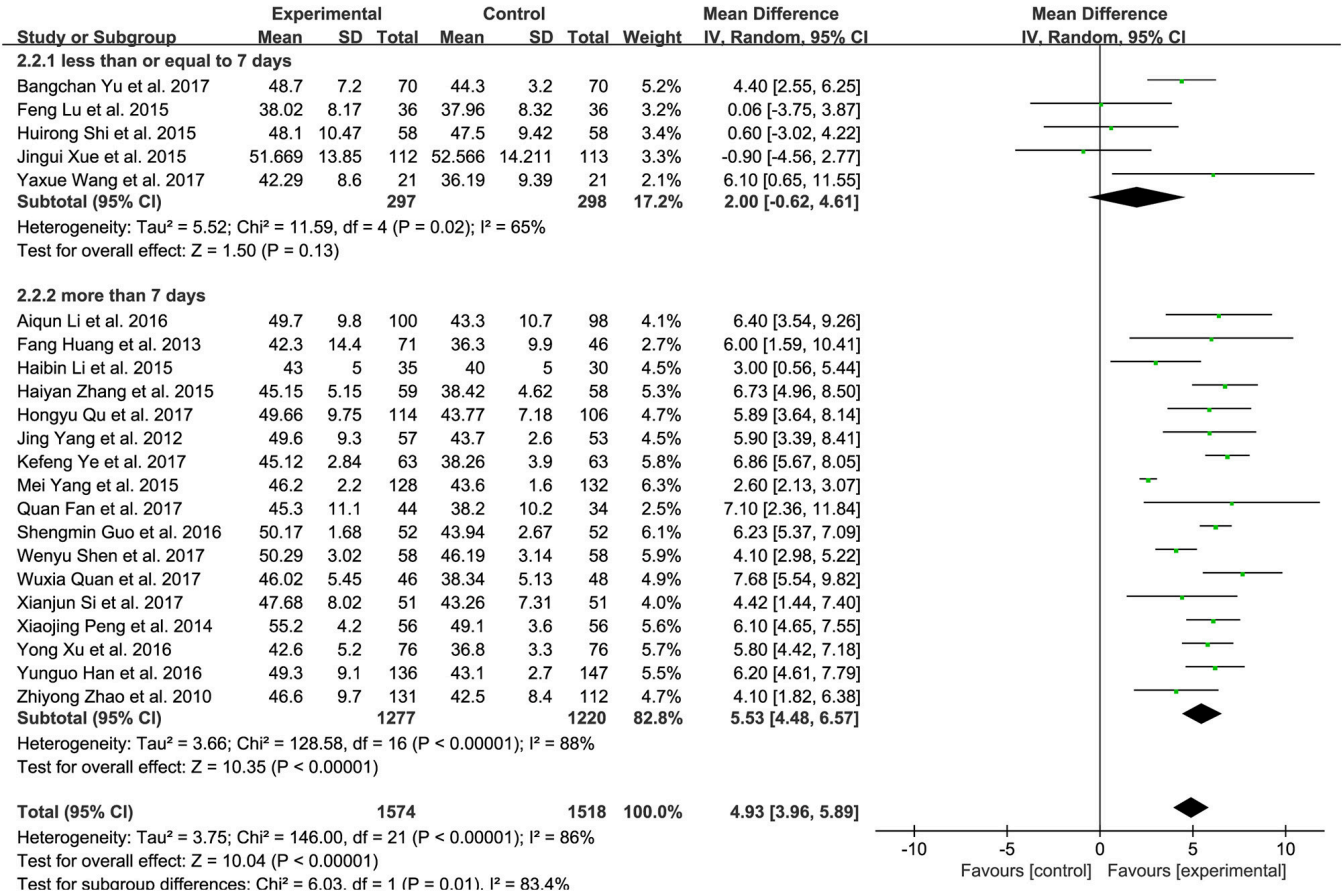
Subgroup analysis of treatment course of XMLI plus western conventional treatment vs. western conventional treatment alone based on LVEF. *I*^2^ and *P* are the criterion for the heterogeneity test, 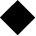 pooled mean difference, —
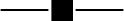
— mean difference and 95% CI. XMLI, Xinmailong Injection; LVEF, left ventricular ejection fraction; MD, mean difference; CI, confidence interval.

There was substantial heterogeneity among these 26 trials. Subgroup analysis of treatment course based on LVEF was further performed to examine whether the length of treatment could contribute to the heterogeneity. Five trials with 595 patients were found to treat CHF patients for less than or equal to 7 days (Lu et al., 2015; Shi H. R. et al., 2015; Xue et al., 2015; Wang, 2017; Yu et al., 2017). Seventeen studies involving 2,497 patients for over 7 days (Zhao et al., 2010; Yang et al., 2012; Huang et al., 2013; Peng et al., 2014; Li and Li, 2015; Yang M. et al., 2015; Zhang, 2015; Guo and Ren, 2016; Han and Guo, 2016; Li et al., 2016; Xu and Xu, 2016; Fan et al., 2017; Quan and Miu, 2017; Qu et al., 2017; Shen et al., 2017; Si, 2017; Ye et al., 2017). Significant difference was observed between these two treatment courses (*P* = 0.01, *I*^2^ = 0%). Long treatment course (over 7 days) (MD 5.53, 95% CI 4.48 to 6.57, *P* < 0.00001, Figure 5) presented higher improvement in LVEF than short one (no more than 7 days) (MD 2.00, 95% CI−0.62 to 4.61, *P* = 0.13).

A sensitivity analysis was performed as the LVEF of XMLI for conventional therapy alone was higher than that of XMLI plus conventional therapy (Xue et al., 2015). In parallel with the above result, LVEF in the experimental group was significantly higher than that of control group (MD 5.13, 95% CI 4.17 to 6.09, *P* < 0.00001).

### Brain natriuretic peptide (BNP) and N-terminal pro-brain natriuretic peptide (NT-proBNP)

**Figure 6:**
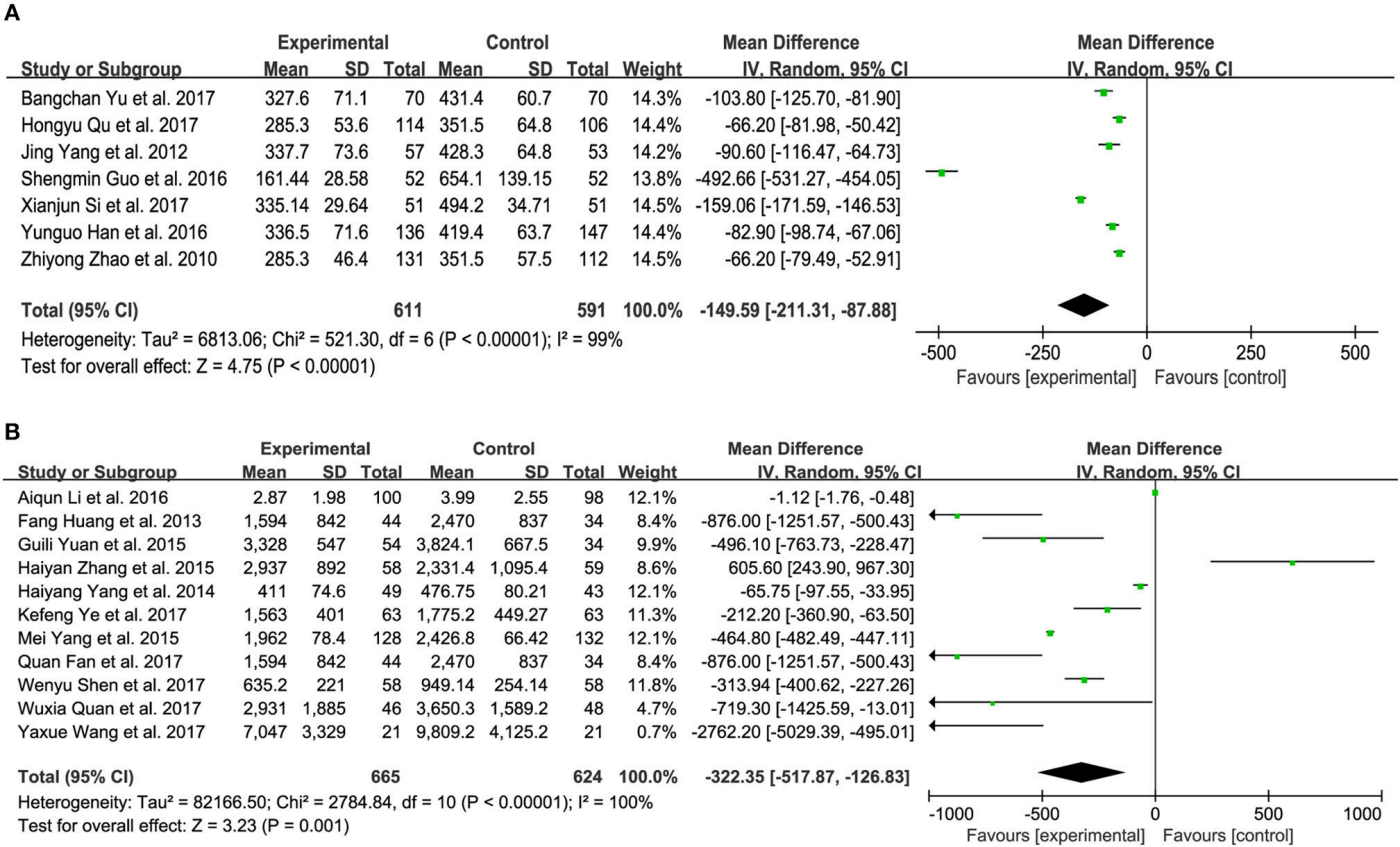
Forest plot of serum BNP **(A)** and NT-proBNP **(B)** of XMLI plus conventional therapy vs. conventional therapy alone for CHF treatment*. I*^2^ and *P* are the criterion for the heterogeneity test, 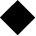 pooled mean difference, —
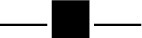
— mean difference and 95% CI. BNP, brain natriuretic peptide; NT-proBNP, N-terminal pro-brain natriuretic peptide; XMLI, Xinmailong injection, CHF, chronic heart failure, CI, confidence interval.

Nine trials measured the BNP level of patients with CHF between XMLI plus conventional therapy and conventional therapy alone. Among these 9 trials, 3 (Yang et al., 2012; Qu et al., 2017; Yu et al., 2017) and 4 (Zhao et al., 2010; Guo and Ren, 2016; Han and Guo, 2016; Si, 2017) trials accessed BNP with the unit of ng/L and pg/mL, respectively, with the rest two adopting the unit of ng/mL and pg/L (Lu et al., 2015; Shi H. R. et al., 2015). As shown in Figure 6A, there was substantial heterogeneity (*I*^2^ = 99%, *P* < 0.00001). Therefore, a random-effects model was used to pool this meta-analysis. In comparison with conventional therapy, XMLI plus conventional therapy could significantly decrease serum BNP in CHF patients (MD − 149.59, 95% CI − 211.31 to − 87.88, *P* < 0.00001, Figure 6A).

Twelve trials assessed the serum NT-proBNP of CHF patients, 5 (Huang et al., 2013; Zhang, 2015; Fan et al., 2017; Quan and Miu, 2017; Ye et al., 2017) and 6 (Yang et al., 2014; Yang M. et al., 2015; Yuan et al., 2015; Li et al., 2016; Shen et al., 2017; Wang, 2017) of which measured with the unit of ng/L and pg/mL, respectively, 1 of which adopted ng/mL (Wu, 2015). Random-effects models were performed because of considerable heterogeneity (*I*^2^ = 100%, *P* < 0.00001). The treatment of XMLI plus conventional therapy could markedly decrease this parameter in CHF patients when compared with conventional treatment (MD−322.35, 95% CI −517.87 to −126.83, *P* = 0.001, Figure 6B).

### Left ventricular end-diastolic dimension (LVEDD)

**Figure 7:**
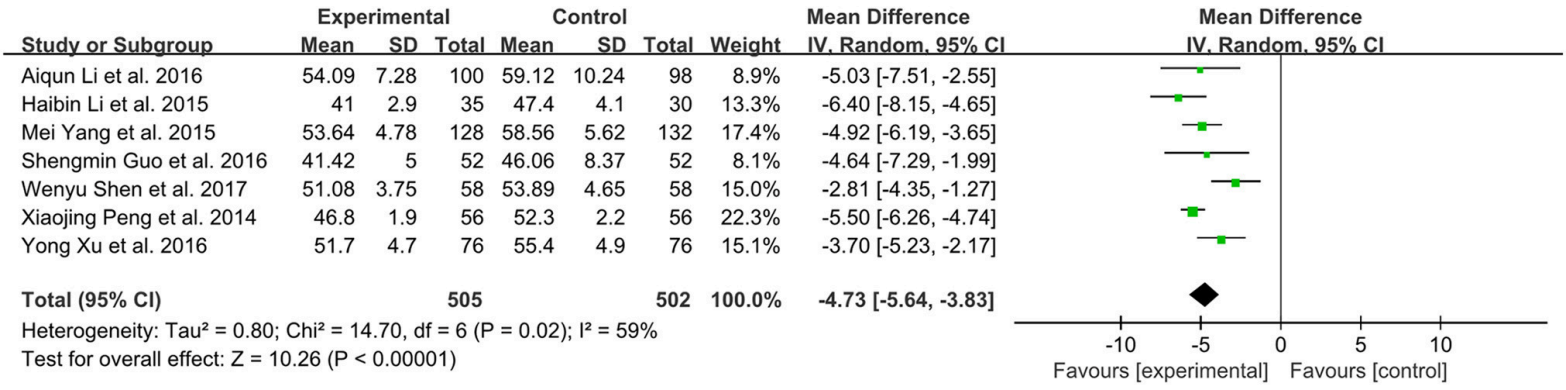
Forest plot of LVEDD of XMLI plus conventional therapy vs. conventional therapy alone for CHF treatment. *I*^2^ and *P* are the criterion for the heterogeneity test, 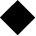 pooled mean difference, —
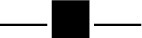
— mean difference and 95% CI. LVEDD: left ventricular end-diastolic dimension; XMLI: Xinmailong injection, CHF: chronic heart failure, CI: confidence interval.

Seven studies with 1007 subjects assessed the levels of LVEDD between the experimental and control group (Peng et al., 2014; Li and Li, 2015; Yang M. et al., 2015; Guo and Ren, 2016; Li et al., 2016; Xu and Xu, 2016; Shen et al., 2017). There was substantial heterogeneity among these trials (*I*^2^ = 59 %, *P* = 0.02). Meta-analysis was performed using a random-effect model. XMLI combined with conventional therapy could significantly reduce LVEDD when compared to conventional treatment alone (MD − 4.73, 95% CI − 5.64 to − 3.83, *P* < 0.00001, Figure 7).

### Six-minutes walking distance (6-MWD)

**Figure 8:**
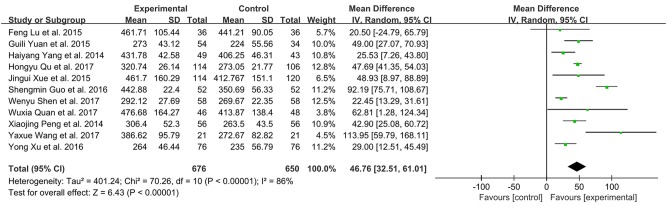
Forest plot of 6 MWD of XMLI plus conventional therapy vs. conventional therapy alone for CHF treatment. *I*^2^ and *P* are the criterion for the heterogeneity test, 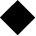 pooled mean difference, —
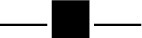
— mean difference and 95% CI. 6 MWD:, 6-min walking distance; XMLI, Xinmailong injection; CHF, chronic heart failure; CI, confidence interval.

There were 11 studies with 1326 subjects reporting 6-MWD between XMLI plus conventional therapy vs. conventional therapy alone. Heterogeneity between these two studies was considerable (*I*^2^ = 86%, *P* < 0.00001). Hence, a random-effect model was used to pool the meta-analysis. XMLI plus conventional therapy achieved a greater improvement when compared with conventional therapy (MD 46.76, 95% CI 32.51–61.01, *P* < 0.00001), which suggested that XMLI was able to increase the exercise tolerance of conventional therapy for CHF treatment (Figure 8).

### Publication bias

**Figure 9:**
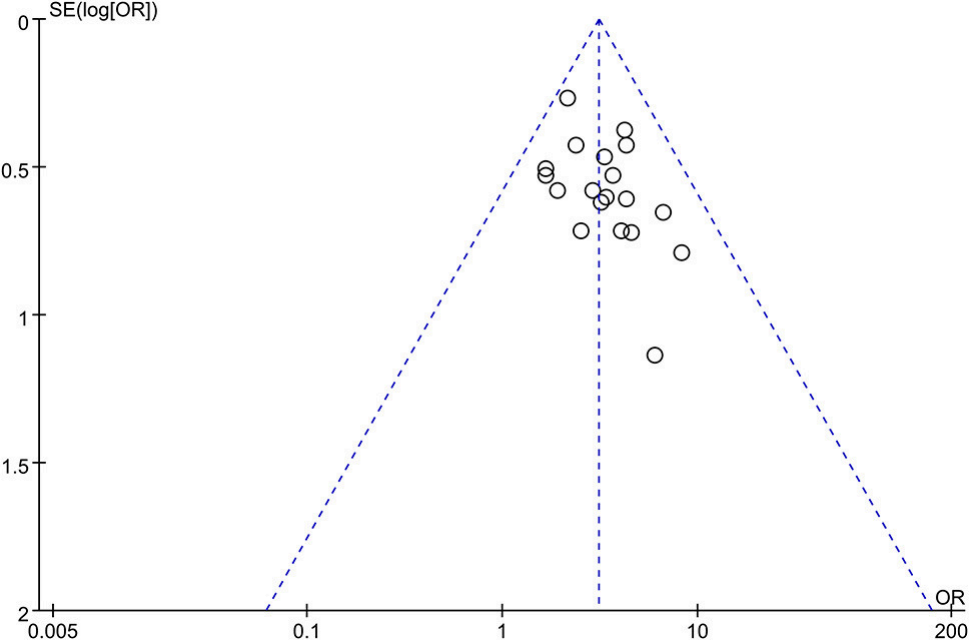
Funnel plot for the publication bias of total effective rate.

A funnel plot was used to evaluate the publication bias. A total of 19 trails were involved in the funnel plot of the total effective rate. No significant asymmetry was observed (Figure 9).

### Adverse events

Eighteen of the included trials investigated the adverse effects (Zhao et al., 2010; Huang et al., 2013; Lu et al., 2013, 2015; Peng et al., 2014; Wu, 2015; Xue et al., 2015; Yang M. et al., 2015; Yuan et al., 2015; Zhang, 2015; Guo and Ren, 2016; Li et al., 2016; Quan and Miu, 2017; Qu et al., 2017; Si, 2017; Wang, 2017; Ye et al., 2017; Yu et al., 2017). Ten of these studies reported that there were no adverse events in terms of blood routine, urine routine, liver function or renal function in both groups (Lu et al., 2013, 2015; Peng et al., 2014; Yuan et al., 2015; Zhang, 2015; Guo and Ren, 2016; Quan and Miu, 2017; Qu et al., 2017; Si, 2017; Yu et al., 2017).

As shown in Table 2, the remaining 8 studies involving 1,274 participants (661 and 613 cases for experimental and control group, respectively) reported adverse effects. As found, XMLI plus conventional treatment caused cutaneous pruritus (9 cases), palpitation (6 cases), light-headed (7 cases), headache (2 cases), hypokalemia (1 case), abnormal liver function (3 cases), major adverse cardiac event (3 cases), while conventional therapy induced leukocytosis (1 case), light-headed (1 case), palpitation (3 cases), dizziness (1 case), light-headed (2 cases), vomiting (1 case), abnormal liver function (2 cases), headache (3 cases), major adverse cardiac event (9 cases) (Zhao et al., 2010; Huang et al., 2013; Wu, 2015; Xue et al., 2015; Yang M. et al., 2015; Li et al., 2016; Wang, 2017; Ye et al., 2017). The incidence rate of adverse effect in the experimental group (4.7%) was slightly higher than that in control group (3.4%). The types of adverse effect nearly equaled between these two groups (7 and 8). The symptoms, such as palpitation, light-headed, headache, and dizziness, were relieved through rest or slowly dropping. None of these adverse events were serious and no included trials reported death during the scheduled treatment.

**Table 2:** The incidence rate of adverse effect.

**Type**	**The number of adverse effect**		**References**
	**Experimental group**	**Control group**	
Cutaneous pruritus	9	0	Huang et al., 2013; Xue et al., 2015; Yang M. et al., 2015
Palpitation	6	3	Yang M. et al., 2015; Li et al., 2016; Ye et al., 2017
Light-headed	7	1	Zhao et al., 2010; Huang et al., 2013; Wu, 2015
Headache	2	3	Ye et al., 2017
Dizziness	0	1	Yang M. et al., 2015
Hypokalemia	1	0	Ye et al., 2017
Abnormal liver function	3	2	Ye et al., 2017
Major adverse cardiac event	3	9	Wang, 2017
Leukocytosis	0	1	Xue et al., 2015
Vomiting	0	1	Ye et al., 2017
Total event	31/661	21/613	–
Incidence rate	4.7%	3.4%	–

## Discussion

### Summary of evidence

In this review, XMLI in all the included studies were from Yunnan Teng yao Pharmaceutical Co., Ltd. The dosages of XMLI were 4/6 mL twice a day or 8 mL per day or 5–10 mg/kg twice a day via intravenous drip. The treatment course varied from 5 to 15 days. The combination of XMLI and conventional therapy displayed better therapeutic effects than conventional treatment alone based on the total effective rate, suggesting that XMLI could improve the clinical efficacy of conventional therapy for the treatment of CHF. The mechanism could contribute to the inhibition of the phosphorylation of ERK1/2, AKT, GSK3β, and protein expression of GATA4 (Qi et al., 2017).

As to left ventricular structure and function, the meta-analysis demonstrated that additional XMLI therapy was superior to conventional treatment, evidenced by increase of LVEF and decrease of LVEDD. In parallel, the combined use of XMLI and conventional therapy was found to increase 6-MWD, the indicator of functional capacity. Both BNP and NT-proBNP levels are good serum markers for evaluating the improvement of heart failure because there is a good correlation between their levels and the severity of heart failure (Jourdain et al., 2007; Oremus et al., 2014). Benefits of XMLI treatment were shown on the decline of serum BNP and NT-proBNP.

Eighteen out of twenty-six studies (69.2%) reported adverse events. Our systematic review observed that the incidence rate of adverse events in the experimental group was a bit higher than that of control group. XMLI plus conventional treatment mainly contributed to cutaneous pruritus, palpitation, light-headed, abnormal liver function and major adverse cardiac event. Similarly, conventional therapy caused palpitation, abnormal liver function, headache and major adverse cardiac event. Rest or slowly dropping was able to relive the symptoms of palpitation, light-headed, headache and dizziness. No serious adverse event was observed in these two groups. XMLI seemed generally safe, but the evidence was too limited to make a decisive conclusion on safety.

Although there was no significant difference on the adverse events between XMLI plus conventional treatment and conventional treatment, additional use of XMLI ameliorated the clinical efficacy and improvement of left ventricular structure and function as well as functional capacity. Therapeutic effects and safety of XMLI remains to be investigated due to a limited number of trials and poor methodological quality of the included trials.

### Strengths and limitations

CHF is a global health problem with 26 million people suffering. Even if diuretics, ACEI, β-blockers, and digitalis are commonly recommended for CHF patients by ESC and ACC/AHA guidelines (Ponikowski et al., 2016; Writing Committee et al., 2016). It is unsatisfying for the western medicine alone on the treatment of CHF due to adverse reactions such as arrhythmia, nausea and impairment of vision caused by digitalis, and dry cough by ACEI (Vegter and de Jong-van den Berg, 2010; Agarwal and Amsterdam, 2015). XMLI has been proven to effectively treat various kinds of failing heart. However, a large number of trials concerning XMLI were conducted independently. This meta-analysis was the first study to systematically evaluate the effects of combined use of XMLI and conventional therapy for CHF treatment and provided preliminary evidence.

However, there still existed limitations in this meta-analysis as followed. Firstly, randomization is necessary to avoid selection bias. Only 6 studies (23.1%) provided specific information on how the random allocation was generated and 2 trials (7.7%) mentioned blinding of participants and personnel and outcome assessment. One article even reported with a high risk in incomplete outcome. For allocation concealment and other bias, no trial mentioned, which could induce exaggerated estimation of therapeutic effect of XMLI plus conventional therapy vs. conventional therapy alone. Thus, the included trials were thought to be generally low quality.

Secondly, we comprehensively searched English and Chinese databases to obtain the eligible articles. All the included studies (26) were published in Chinese. One trial in English was searched but excluded due to its data could not combine with other selected studies (Ma et al., 2013). Thus, the information about RCTs of XMLI was unclear in other countries or populations.

Thirdly, the included trials used different diagnostic criteria separately or combinatively for patients with CHF (Table 1). Participants with CHF also had different indications. Twelve of twenty-six studies mentioned the different indications, such as coronary heart disease, dilated cardiomyopathy, hypertensive heart disease, and rheumatic heart disease (Yang et al., 2012, 2014; Huang et al., 2013; Li and Li, 2015; Lu et al., 2015; Yang M. et al., 2015; Yuan et al., 2015; Guo and Ren, 2016; Han and Guo, 2016; Fan et al., 2017; Quan and Miu, 2017; Wang, 2017). But these CHF patients were not divided into subgroup of CHF before or after the treatment of XMLI plus conventional therapy or conventional therapy alone. Only three trials reported the single indication for CHF patients: coronary atherosclerotic heart disease (Shi H. R. et al., 2015), dilated cardiomyopathy (Lu et al., 2013) and coronary heart disease (Shen et al., 2017). It was unclear for the rest 11 trials in terms of the precision indication except for CHF.

Fourthly, meta-analysis results may be influenced by the treatment course. In this review, the courses were 5 (7 studies), 7 (1 studies), 10 (4 studies), 14 (11 studies), and 15 (2 studies) days. One article reported 7–14 days based on disease severity (Yang M. et al., 2015). Continued follow-up after the treatment period was necessary to investigate the long-term treatment effect. However, only three studies performed this: one evaluated the clinical effect after 2 weeks (Lu et al., 2015), one after 6 weeks (Peng et al., 2014) and one after 1 and 3 months (Wang, 2017). While the rest assessed immediately after the termination of the treatment period.

Fifthly, seven outcome measurements, including the total effective rate, LVEF, BNP, NT-proBNP, LVEDD, 6 MWD, and adverse effect, were used to evaluate the effect of additional XMLI on CHF treatment. In fact, other outcome measures, such as high sensitivity C reactive protein (hsCRP), heart rate (HR), cardiac troponin I (CTnI), all-cause mortality, hospitalization, left ventricular end-systolic/diastolic volume, NYHA functional class, and blood pressure, are good indexes for the assessment of cardiac function on CHF patients. However, these indexes were only reported in a limited number of trials, making it nearly impossible to pool these data.

Sixthly, in the included trials, there was no precise description of medication in the control group. These trials just mentioned “conventional treat RCT ment” or “standard therapy,” which indicated that ACEIs, beta-blockers, MRAs, diuretics and ARBs could be used in the control group varied from person to person according to CHF patients' signs and symptoms. Precisely, the impacts of which type of western medicine in combination with XMLI are suggested to investigate in RCTs.

Lastly, drug safety is essential in the development of alternative medicines for health care. Eighteen of the included 26 studies investigated adverse events. High-quality and large scale RCTs, with comprehensive adverse events recorded, are still needed to evaluate the impact of XMLI treatment on CHF patients.

## Conclusion

The treatment of CHF has been a worldwide challenge. The traditional Chinese medicine XMLI plus conventional treatment may exert beneficial effects to improve cardiac function of patients with CHF. XMLI was therefore suggested to be taken into account during the conventional treatment of CHF. It is worth noticing the limitations in this review. The efficacy and safety of XMLI as an adjuvant treatment for CHF still need methodologically rigorous trials to verify.

## Author contributions

YZ, XX, and XL put forward this topic and designed this review. XL, LZ, HZ, and YY performed article screening, data collection and extraction, and manuscript writing. JW, HoL, HaL, and RW conducted the data analysis. JW, SW, and XZ polished the written English.

### Conflict of interest statement

The authors declare that the research was conducted in the absence of any commercial or financial relationships that could be construed as a potential conflict of interest.
